# Prospects and challenges of traditional Chinese medicine supramolecular nano-drug delivery systems in the treatment of cerebral ischemia

**DOI:** 10.3389/fphar.2026.1917854

**Published:** 2026-09-11

**Authors:** Xinning Ren, Ru Qiao, Can Liu, Lei Shen, Wenjun He, Yinan Wang, Xue Pan, Fuyuan He

**Affiliations:** 1 College of Pharmacy, Hunan University of Chinese Medicine, Changsha, China; 2 Hunan Provincial Key Laboratory of Drugability and Preparation Modification of TCM, Hunan University of Chinese Medicine, Changsha, China; 3 Laboratory of Supramolecular Mechanism and Mathematical Characterization of Traditional Chinese Medicine, Hunan University of Chinese Medicine, Changsha, China

**Keywords:** blood-brain barrier, cerebral ischemia, drug delivery, microenvironment responsiveness, modernization of Chinese medicine, self-assembly, traditional Chinese medicine supramolecules

## Abstract

Ischemic stroke is a leading cause of permanent disability and death worldwide. Effective neuroprotective therapy is hindered by two major obstacles: the blood-brain barrier and the complex pathological cascades triggered by reperfusion injury. Supramolecular traditional Chinese medicine (STCM) refers to nano-aggregates that spontaneously form during herbal decoction. These natural assemblies offer several advantages for brain-targeted delivery, including biocompatibility, structural adaptability, multi-component synergy, and responsiveness to the lesion microenvironment. This review begins by examining the pathological microenvironment of the ischemic penumbra. In this region, acidosis, elevated reactive oxygen species, and upregulated matrix metalloproteinases act as endogenous stimuli triggering the disassembly of STCM nanostructures and the release of drugs at the site of injury. We then trace the development of TCM-based nanocarriers and describe four BBB-crossing mechanisms that are unique to STCM. These include glycan-mediated self-targeting through multivalent surface interactions, enhanced permeation through co-assembly with aromatic resuscitation herbs, reversible disassembly of non-covalent networks in response to pathological signals, and functional synergy among paired herbal components. We also summarize preclinical evidence for the use of STCM in ischemic stroke therapy, synthesizing data from representative active ingredients and herb pairs. Finally, we evaluate translational bottlenecks and propose strategic research directions. This work establishes a unified theoretical and methodological foundation for designing STCM-based precision brain-targeted therapeutics against cerebral ischemia.

## 1 Introduction

Ischemic stroke (IS) is defined as ischemic and hypoxic injury of brain tissue caused by interruption of cerebral blood flow. It accounts for approximately 70%–80% of all stroke cases (Yang et al., 2025) and is characterized by high morbidity, mortality, and disability (Peng, 2007). Current clinical treatments for acute IS, including intravenous thrombolysis and mechanical thrombectomy (Powers et al., 2018; Rabinstein, 2020), are limited by a narrow therapeutic time window and the risk of exacerbated injury due to oxidative stress and inflammatory cascades post-reperfusion. The blood–brain barrier (BBB) significantly prevents about 98% of small-molecule drugs and almost all macromolecular therapeutics from reaching effective concentrations in the brain (Long and Xiao, 2006).

Although nano-drug delivery systems employ strategies such as receptor-mediated and adsorptive-mediated transcytosis to cross the BBB (Mu et al., 2022), synthetic nanocarriers are generally limited by low drug loading capacity, immunogenicity risks, and high translation costs. Furthermore, the long-term safety of most synthetic materials *in vivo* remains unclear (Jiang et al., 2025). Supramolecular traditional Chinese medicine (STCM) refers to nano-aggregates spontaneously formed during water decoction, assembled through weak non-covalent interactions, including hydrogen bonds, π–π stacking, and hydrophobic effects. Due to their natural origin, self-carrying capability, and multi-component synergy, STCM aggregates offer distinct advantages for crossing the BBB (Li et al., 2021).

Existing reviews on TCM-based nano-delivery systems have focused mainly on encapsulating single natural product monomers using synthetic or semi-synthetic carriers (Mu et al., 2022; Liu S et al., 2025). To date, however, no review has systematically addressed the carrier-free, multi-component supramolecular aggregates that form naturally in herbal decoctions. In particular, their BBB-permeation behavior, the structural basis of their synergistic effects, and the supramolecular properties of herb pairs remain poorly understood. This review integrates the meridian-guiding theory of TCM with supramolecular self-assembly mechanisms to elucidate the emergent, multi-pathway synergistic BBB-crossing mechanisms of herb-pair co-assemblies. We aim to address the theoretical gap in supramolecular TCM for precision-targeted delivery in cerebral ischemia, providing a novel perspective to overcome current efficacy limitations (Li et al., 2024). Rather than providing a broad overview, this review compares the BBB-crossing mechanisms of STCM with those of synthetic nanocarriers in the context of ischemic stroke pathophysiology. We review available preclinical evidence, identify key translational barriers, and suggest actionable directions for future research.

## 2 Pathophysiology of the BBB during cerebral ischemia

### 2.1 Dynamic opening-closing changes of the BBB during ischemia

The BBB is a central component of the neurovascular unit. It consists of brain microvascular endothelial cells (BMECs), pericytes, astrocytic end-feet, and an extracellular basement membrane (Stevenson et al., 1986). Under physiological conditions, BMECs form a highly selective physical barrier via tight junction proteins, including Claudin-5, Occludin, and ZO-1. They also express efflux transporters such as P-glycoprotein (P-gp) and breast cancer resistance protein (BCRP). Together, these components strictly limit the entry of exogenous substances into the brain (Liu et al., 2024; Liu S et al., 2025).

**FIGURE 1:**
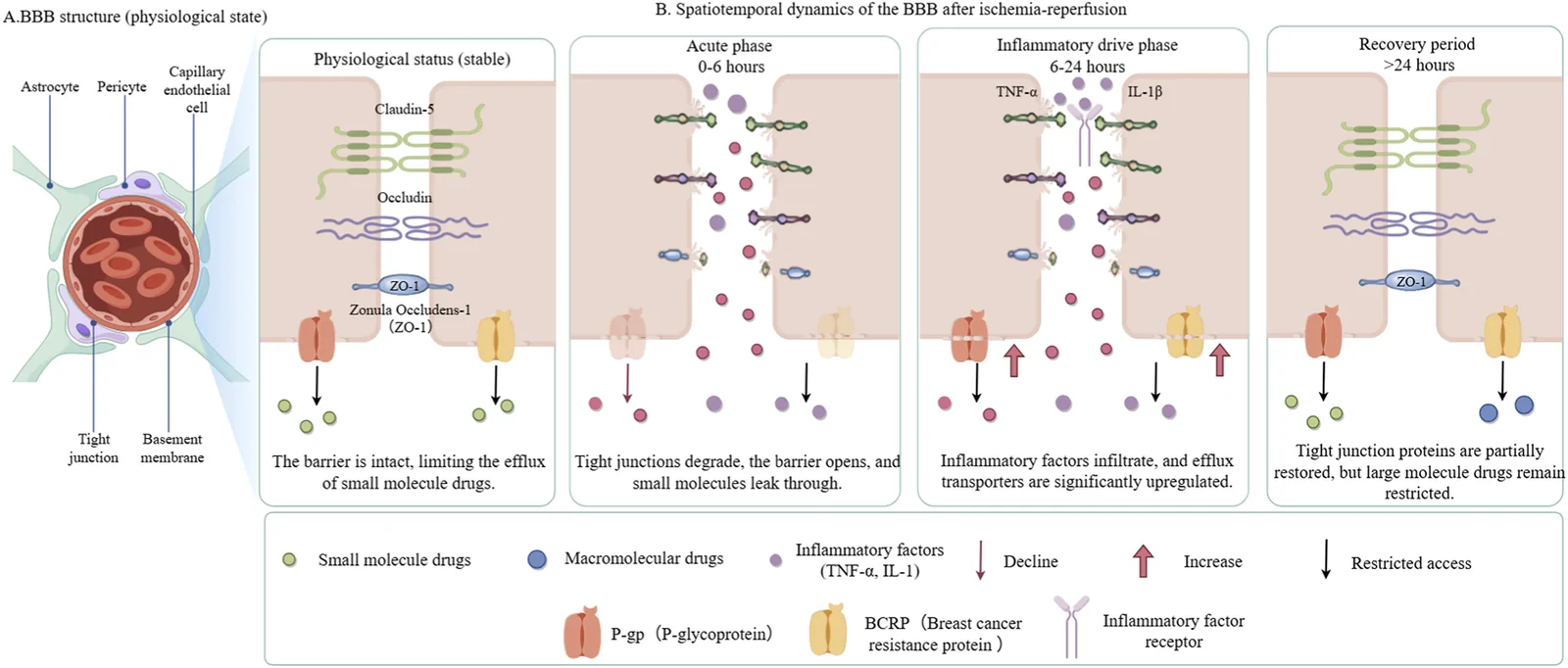
Physiological structure of the BBB and its spatiotemporal dynamic changes after ischemia-reperfusion. **(A)** Ultrastructural schematic of the BBB under physiological conditions. The BBB is composed of astrocytes, pericytes, capillary endothelial cells, the basement membrane, and tight junctions between adjacent endothelial cells. **(B)** Four-phase temporal evolution of BBB integrity after cerebral ischemia-reperfusion injury. (i) Stable phase: maintenance of normal barrier function. (ii) Acute phase (0–6 h): degradation of tight junction proteins (Claudin-5, Occludin, ZO-1), barrier disruption, and onset of small-molecule extravasation. (iii) Inflammation-driven phase (6–24 h): extensive infiltration of pro-inflammatory cytokines (TNF-α, IL-1β), further disruption of barrier integrity, and dysregulation of efflux transporters (P-gp, BCRP), characterized by variable upregulation or downregulation, collectively establishing a complex pathological microenvironment. (iv) Recovery phase (more than 24 h): partial restoration of tight junction protein expression.

Following cerebral ischemia, BBB integrity is disrupted in a spatiotemporally heterogeneous manner. In the ischemic core, energy depletion leads to altered phosphorylation of tight junction proteins, redistribution and degradation of Occludin and Claudin-5, and a significant increase in BBB permeability within hours after reperfusion (Wakayama et al., 2022). At the same time, efflux transporter function becomes disturbed. P-gp and BCRP expression and activity decline in the early phase of ischemia because of ATP depletion. Later, pro-inflammatory cytokines such as TNF-α and IL-1β drive compensatory overexpression of these transporters, creating a temporary efflux enhancement window (Zhao Y et al., 2022). This spatiotemporal switching mode requires STCM to possess dual adaptability. It can rapid entry via passive diffusion during the acute open phase, and a switch to active, targeted translocation during the barrier repair phase (Figure 1).

Consequently, designing supramolecular nano-delivery systems for cerebral ischemia requires precise targeting of the time window of BBB permeability.

### 2.2 Microenvironmental characteristics of the ischemic penumbra: low pH, high ROS, and MMP upregulation

The ischemic penumbra refers to the region surrounding the ischemic core, where blood flow is reduced but the tissue remains viable. It represents the golden target for neuroprotective therapy (Ermine et al., 2021). This region exhibits a unique pathological microenvironment, with characteristic parameters functioning as responsive drug-release switches for supramolecular nanosystems (Wu et al., 2024; Li et al., 2026). Recently, microenvironment-responsive nano-delivery systems for cerebral ischemia have become an area of intense research interest, centered on the principle of using pathological signals to trigger precise drug release.

#### 2.2.1 Extracellular acidification

Ischemia-hypoxia enhances anaerobic glycolysis, leading to substantial lactate accumulation. Extracellular pH in the penumbra can drop from physiological 7.35–7.45 to 6.5–6.8 or lower (Peek et al., 1989). This mildly acidic environment is an ideal trigger for disrupting hydrogen bond networks, electrostatic interactions, as well as acetal and hydrazone bonds within supramolecular assemblies (Tóth et al., 2020). Many TCM active ingredients, such as flavonoids and saponins, contain phenolic hydroxyl or carboxyl groups. Under neutral conditions, they form stable nano-aggregates via intermolecular hydrogen bonds; in the acidic microenvironment, these networks rearrange or disintegrate, thereby enabling pathological site-specific drug release (Sun et al., 2024).

#### 2.2.2 Reactive oxygen species (ROS) burst

Following ischemic stroke, a massive ROS burst occurs in brain tissue, activating apoptotic pathways and exacerbating neuronal damage (Allen and Bayraktutan, 2009; Jin et al., 2010; Shigemoto-Mogami et al., 2018). ROS levels in the penumbra can reach 5–10 times physiological baselines and persist for days (Wang D et al., 2025). The high-ROS environment creates optimal conditions for activating ROS-responsive drug delivery systems. For instance, supramolecular nanoparticles containing thioketal, oxalate ester, or phenylboronic ester bonds undergo structural dissociation upon ROS exposure to release encapsulated drugs (Jian et al., 2025; Chen Q et al., 2026). Integrating TCM ingredients possessing intrinsic antioxidant activity into ROS-responsive supramolecular systems can yield a cascade effect of ROS scavenging, drug release, and synergistic protection (Li et al., 2025).

#### 2.2.3 Matrix metalloproteinase (MMP) upregulation

MMP-2 and MMP-9 are significantly induced after cerebral ischemia, peaking 12–72 h post-reperfusion (Krizanac-Bengez et al., 2006). MMPs degrade collagen IV and laminin within the BBB basement membrane, disrupting BBB integrity (Rosenberg et al., 1998), and function as activation targets for enzyme-responsive nano-delivery systems. MMP-specific substrate peptides such as GPLGVRG, incorporated into supramolecular systems, can be cleaved by MMPs at the ischemic site, triggering nanoparticle disassembly or surface charge reversal to facilitate deep tissue penetration (Liu Q. et al., 2025).

In summary, the dynamic opening-closing properties of the BBB and the low-pH, high-ROS, high-MMP microenvironment of the penumbra present a spatiotemporally heterogeneous pathological target. An ideal drug delivery system should navigate this complex environment by remaining stable in the bloodstream and releasing drugs responsively upon reaching the lesion. STCM nanosystems, owing to the reversibility and environmental sensitivity of their non-covalent assemblies, inherently possess a response logic that aligns with the above pathological features.

## 3 Brain-targeting strategies of TCM-based nano-delivery systems

### 3.1 Inherent limitations of synthetic nanosystems for brain targeting

**FIGURE 2:**
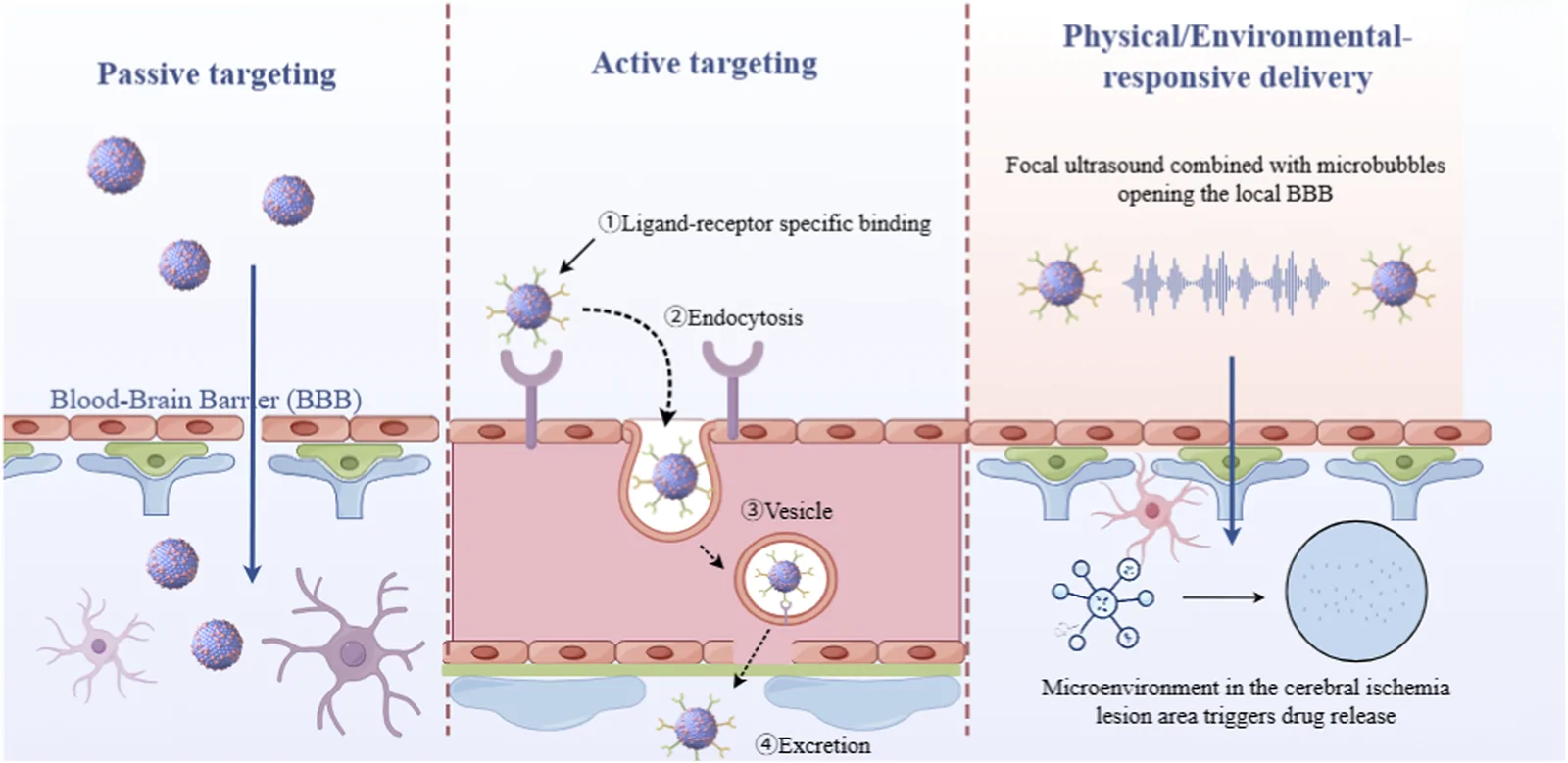
Targeting mechanisms of nanoparticles across the BBB. Schematic illustration of the primary mechanisms of targeted drug delivery across the BBB. Passive targeting: appropriately sized drug-loaded nanoparticles can extravasate through the compromised or leaky BBB into the ischemic cerebral lesion via passive diffusion and the enhanced permeability and retention effect. Active targeting: nanoparticles surface-modified with targeting ligands bind specifically to receptors on brain capillary endothelial cells, followed by a sequential cascade of (i) ligand-receptor specific recognition, (ii) endocytosis, (iii) vesicular intracellular trafficking, and (iv) exocytosis, thereby achieving efficient transcellular transport across the BBB. Physical disruption of the BBB for enhanced delivery: focused ultrasound combined with microbubbles is employed to noninvasively and reversibly open the BBB locally through sonoporation, enabling rapid and transient accumulation of therapeutic agents. Pathological microenvironment-responsive drug release: intelligent stimuli responsive nano-delivery systems are designed to trigger on-demand drug release in response to the distinctive pathological cues of the ischemic lesion, including acidic pH, elevated ROS, and upregulated specific enzymes.

Synthetic nano-delivery systems represent the most mature direction in brain-targeting research, categorized into passive targeting, active targeting, physical or environmentally responsive delivery (Figure 2). However, clinical translation faces three inherent bottlenecks.

First, active targeting strategies rely on chemical conjugation of specific ligands such as transferrin, lactoferrin, or Angiopep-2 (Steffens and Wagner, 2023). This modification increases formulation complexity and introduces immunogenicity risks. For example, fusing a transferrin receptor-targeting single-chain variable fragment with therapeutic antibodies can enhance immunogenicity and trigger the production of anti-drug antibodies. In addition, PEGylation presents its own immunogenic challenges. Anti-PEG antibodies have been detected in both healthy individuals and patients treated with PEGylated drugs. These antibodies can alter nanocarrier biodistribution, induce inflammation, and cause hypersensitivity reactions (Shiraishi, 2025).

Second, the long-term metabolic fate and accumulation-induced toxicity of synthetic carriers (e.g., PLGA, PEGylated liposomes) remain poorly characterized. Biodegradation products of inert materials may precipitate chronic inflammatory responses. PLGA degradation yields lactic and glycolic acid monomers, which are potential inflammatory triggers stimulating pro-inflammatory cytokines (TNF-α, IL-6, TGF-β1) (Chen et al., 2022). Prolonged local retention of nanoparticles may result in toxicity, inflammation, and immune responses. Furthermore, the acidic microenvironment generated by PLGA degradation reduces cell viability, increases apoptosis, and upregulates α-SMA, a marker of myofibroblast activation. In animal models, PLGA implants sustained and exacerbated local inflammatory responses over 12 weeks (Chor et al., 2022).

Third, batch-to-batch consistency of synthetic nanosystems depends on multiple factors, including polymer molecular weight distribution and ligand conjugation efficiency. This makes quality control during scale-up particularly challenging (Zhang M. et al., 2023). Synthesis is highly process-dependent. Without strict control, batch-to-batch variability arises, which compromises the reproducibility of biological modifications. Passive targeting formulations, such as curcumin-loaded solid lipid nanoparticles, while capable of penetrating brain tissue via gaps in the compromised BBB (Kakkar et al., 2013), demonstrate targeting efficiency highly dependent on the time window and degree of BBB disruption. They also suffer from considerable inter-individual variability and poor specificity (Zhang M. et al., 2023).

### 3.2 Implications of biomimetic strategies for supramolecular TCM design

Biomimetic nanosystems camouflage nanomedicines by leveraging the body’s own cells, membrane structures, and vesicles to achieve immune evasion and intrinsic homing. Cell membrane coating technology encapsulates natural cell membranes (such as macrophage, neutrophil, platelet, or stem cell membranes) around synthetic nanoparticle cores, endowing the nanoparticles with the characteristic membrane proteins and homing functions of the source cells. Curcumin-loaded, erythrocyte-membrane-functionalized biomimetic nanoparticles were prepared; these nanoparticles can inhibit Aβ aggregation and reduce its neurotoxicity (Wang et al., 2024). In addition, a study developed a mesoporous silica nanocarrier loaded with the death-related protein kinase 1 (DAPK1) inhibitor TC-DAPK6 and Rhodamine B, which was subsequently coated with macrophage membranes to facilitate its crossing of the blood-brain barrier and achieve selective accumulation at the epileptic focus (Geng et al., 2024). This strategy exhibits negligible immunogenicity and high targeting efficiency; however, the fabrication process is intricate, and maintaining batch-to-batch consistency of membrane protein activity remains challenging.

Although synthetic nanosystems and biomimetic nanosystems each possess their respective advantages and limitations in delivery strategies, both are dependent on exogenous materials or complex biological components. They thus share common bottlenecks in formulation simplification and heterogeneity reduction. STCM occupies a unique position at the intersection of synthetic and biomimetic paradigms. It combines the biomimetic advantages of natural sources, requiring no exogenous carriers and exhibiting low immunogenicity, while also achieving structural controllability through the regulation of decoction conditions. Thus, STCM embodies an integration of natural biomimetics and designability.

## 4 Supramolecular traditional Chinese medicine nano-delivery systems

### 4.1 Evolution of the STCM concept and theoretical framework

Chinese scholars identified STCM in TCM decoctions in the early 2000s (Yi and Xu, 2004; Zhuang et al., 2008; Hu et al., 2009). Researchers have found that self-assembly phenomena occur at multiple stages of TCM, including crude material, processing, decoction, formulation preparation, storage, and final administration in the body (Hu et al., 2022). Furthermore, STCM has been observed in the aqueous extracts of 60 single herbal ingredients and 24 TCM formulas (Zhuang et al., 2008). This discovery challenged active ingredient determinism and opened new paths for understanding TCM holism and synergy. In 2014, Professor He Fuyuan proposed the supramolecular “imprinting template' mechanism of TCM action, elevating the interaction between TCM and the body to the supramolecular level (He et al., 2014). In 2021, Professor Qiao Hongzhi proposed the structural Chinese medicine theory (Qiao et al., 2021), establishing the core idea that components are the basis of efficacy, while structure is the form of action. In 2025, Zhao Junning et al. formally introduced the concept of “formula nano-body', defining it as an ordered aggregate with nanoscale structure and function spontaneously assembled by the inherent active components of TCM through non-covalent interactions during processing, extraction, formulation, and *in vivo* absorption. This provides a systematic theoretical framework for the STCM field (Zhao et al., 2025).

In this review, supramolecular traditional Chinese medicine nano-delivery systems (STCM-NDDS) are defined as nanoscale (1–1,000 nm) ordered aggregates. These aggregates form spontaneously from active TCM ingredients (single herbs, herb pairs, or formulas) through non-covalent interactions during water decoction or simulated *in vivo* processes (Wang et al., 2021). This definition emphasizes two key features, namely, carrier-free and spontaneous assembly. These distinguish STCM-NDDS from conventional TCM nanoformulations that rely on synthetic materials. Such aggregates require no exogenous synthetic carriers and can achieve drug encapsulation, protection, targeted delivery, and responsive release (Li et al., 2025).

Carrier-free self-assembled nanodrugs have attracted considerable attention in recent nanomedicine research, especially in oncology. Researchers have conducted a systematic review of the preparation methods, performance evaluation, and advantages over traditional carrier systems of carrier-free nanomedicines constructed from hydrophobic drug molecules, highlighting the significant potential of carrier-free strategies to increase drug-loading capacity, simplify manufacturing processes, and reduce carrier-related toxicity (Karaosmanoglu et al., 2021). Although the aforementioned review focused primarily on cancer therapy, its core concept of utilizing the inherent self-assembly properties of drug molecules to construct delivery systems is highly consistent with the design philosophy of STCM. This convergence supports the hypothesis that STCM-NDDS, as natural self-carrying supramolecular assemblies, may overcome certain limitations inherent to synthetic nanocarriers.

### 4.2 Superior mechanisms of STCM-NDDS for BBB crossing

**FIGURE 3:**
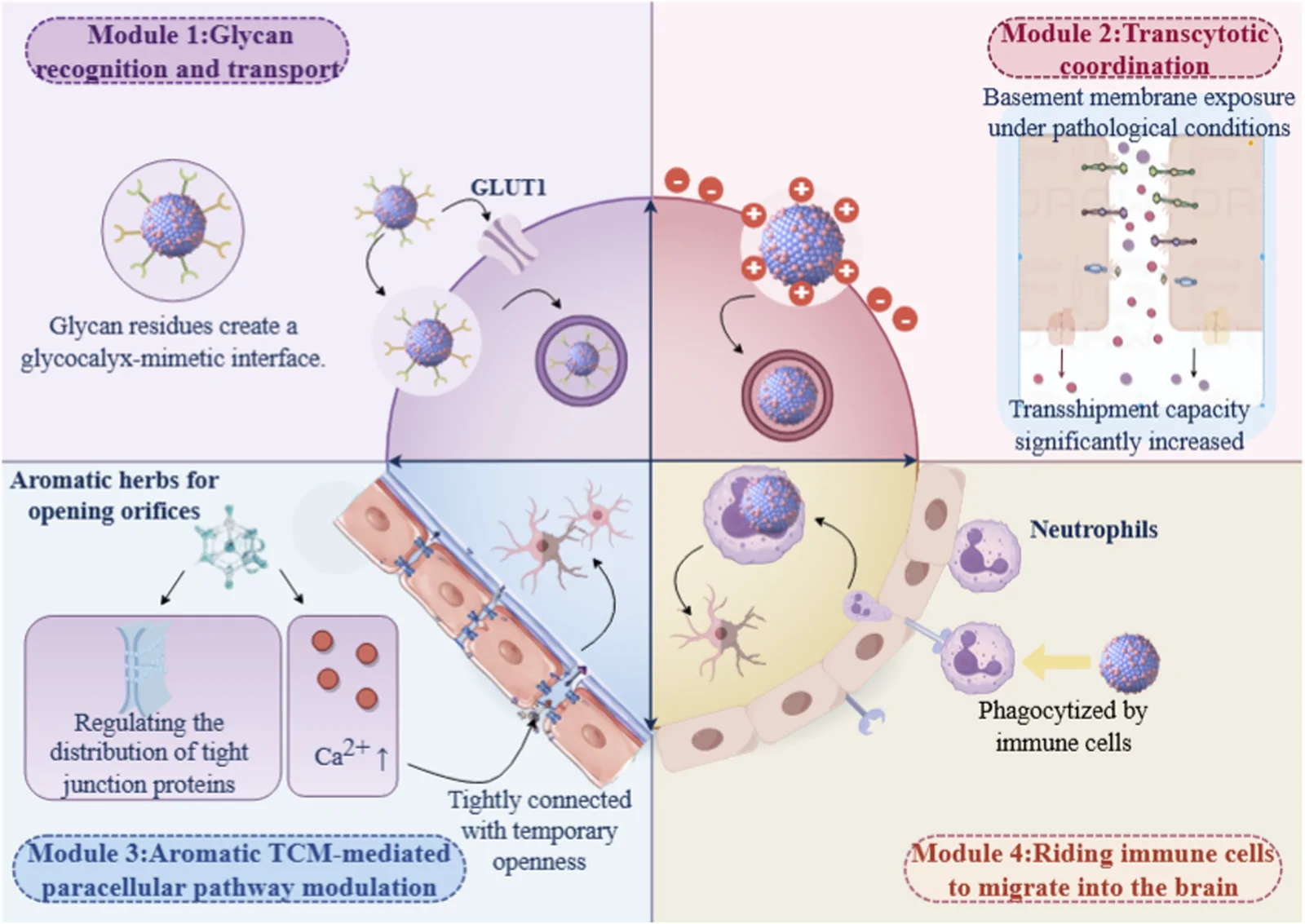
Multiple pathways of STCM-NDDS across the BBB. Schematic illustration of multimodal BBB permeation strategies based on the intrinsic properties of supramolecular traditional Chinese medicine. (Module 1) Glycan-mediated recognition and transport: The sugar chain residues on the surface of the nanocarriers mimic the glycocalyx interface of endothelial cells and promote active transmembrane transport through the glucose transporter 1 (GLUT1) receptor recognition mechanism. (Module 2) Transcytotic coordination: Under pathological conditions with basement membrane exposure, nanoparticles with appropriate surface charge or specific modifications exhibit significantly enhanced endocytic and exocytic activities, thereby increasing transcellular transport flux. (Module 3) Aromatic TCM-mediated paracellular pathway modulation: Typical aromatic resuscitation active ingredients (borneol, muscone) modulate the distribution of tight junction proteins and elevate intracellular Ca^2+^ concentration, resulting in transient inter-endothelial openings and providing a paracellular route for drug transport. (Module 4) Immune cell-mediated hitchhiking for brain migration: Drug-loaded nanoparticles are phagocytosed by immune cells such as neutrophils, and subsequently cross the BBB into the brain parenchyma by leveraging inflammatory chemotactic gradients following cerebral ischemia.

The uniqueness of STCM in crossing the BBB lies in its reliance on the integration of multiple trans-BBB pathways, flexibly switching among them under different pathological phases and microenvironmental conditions (Figure 3).

#### 4.2.1 GLUT1-mediated glycorecognition transport

The luminal membrane of BMECs exhibits high expression of glucose transporter 1 (GLUT1). Astragalus and other Qi-tonifying herbs are rich in polysaccharides; during supramolecular self-assembly, sugar chain residues are exposed on the surface, forming a glycocalyx-like interface recognized by GLUT1, facilitating receptor-mediated transcytosis into the brain along the nutrient pathway (Xia et al., 2023). Mannose-integrated nanoparticles exploit GLUT1 recycling to cross the BBB for multi-target treatment of Alzheimer’s disease (Lei et al., 2024). The polysaccharide groups naturally carried on the surface of STCM function as built-in passports, enabling active transport without exogenous ligand modification.

#### 4.2.2 Adsorptive-mediated transcytosis synergy

Adsorptive-mediated transcytosis is a receptor-independent pathway. It is driven by electrostatic interaction between the positive surface charge of the drug delivery system and anionic microdomains on the BBB endothelial cell luminal membrane. These anionic microdomains include heparan sulfate proteoglycans (Lim et al., 2015; Falanga et al., 2018). This process induces membrane invagination, facilitating nanoparticle internalization and transport to the brain parenchyma (Lu, 2012). Compared with receptor-mediated transcytosis, this pathway is independent of specific receptor expression, maintaining stable barrier-crossing efficiency when receptors are downregulated (Lee et al., 2026). Alkaloid components in STCM, such as tetramethylpyrazine and berberine, confer a positive surface charge, facilitating electrostatic adsorption onto the negatively charged endothelial cell membrane and exposed basement membrane, thereby triggering adsorptive-mediated transcytosis (Arora et al., 2024). Under neuroinflammatory conditions, increased negative charge on the endothelial luminal membrane and exposed basement membrane amplify adsorptive-mediated transcytosis, making it a powerful complement to the GLUT1 pathway, especially in the early stage of BBB dynamic changes.

#### 4.2.3 Natural guiding drug-mediated paracellular regulation

According to the meridian tropism theory of TCM, guiding drugs (yaoyin) direct other herbs toward diseased sites (Dai and Yang, 2018; Li J. et al., 2023). Aromatic resuscitation herbs (borneol, Acorus tatarinowii, musk, storax, benzoin) are well-documented to enhance BBB permeability (Fang et al., 2023). Mechanistically, these compounds trigger transient, reversible tight junction relaxation by remodeling tight junction protein localization and elevating intracellular calcium levels (Lu et al., 2023). When incorporated into STCM aggregates via host-guest inclusion or hydrophobic co-assembly, these permeation-promoting moieties locally dissociate at the BBB microenvironment to generate transient transport channels for the whole supramolecular nanoparticle. This model provides a concrete supramolecular mechanistic explanation for the traditional messenger herb concept.

However, direct empirical evidence distinguishing whether aromatic actives exert biological effects as free standalone molecules or integrated supramolecular subunits remains limited. Published work validates borneol’s BBB-modulating capacity as a pure monomer (Tan et al., 2023; Wang Y. et al., 2025), yet monomeric bioactivity cannot be directly extrapolated to borneol embedded within STCM networks. A key unresolved question is whether borneol integrated into STCM through non-covalent interactions exerts an effect on BBB permeability distinct from that of free borneol. If such a difference exists, it could result from sustained release or from synergistic interactions among multiple components.

#### 4.2.4 Immune cell hitchhiking biomimetic strategy

Macrophages play a dual role in the pathology of cerebral ischemia. By leveraging the inherent pathological site homing ability, strong phagocytic activity, and natural barrier-crossing capacity of immune cells such as macrophages, nanomedicines can be loaded onto these living carriers and actively transported to the lesion area for release, thereby significantly improving drug delivery efficiency and targeting (Zhang et al., 2025). After cerebral ischemia, peripheral monocytes and neutrophils actively migrate into the brain via interactions with the highly expressed adhesion molecules ICAM-1 and VCAM-1 on the BBB. Circulating STCM can be phagocytosed by these immune cells, co-migrate with them across the BBB, and be subsequently released in the lesion area. Macrophage membrane-coated nanoparticles have been used to deliver curcumin to the ischemic brain region by exploiting the natural BBB-crossing ability of macrophages (Han et al., 2026). Amphiphilic nanoparticles based on Angelica sinensis polysaccharides, camouflaged with macrophage membranes, successfully delivered tetramethylpyrazine and ethyl ferulate to the brain injury site (Su et al., 2022).

The parallel operation and synergistic effects of the four pathways endow STCM with unique emergent delivery advantages over synthetic nanoparticles. This advantage stems from the collective behavior formed by the multi-component complex system, which cannot be simply attributed to the effect of any single pathway.

### 4.3 Representative applications of STCM in cerebral ischemia treatment

Compared to the abundant achievements of synthetic nanocarriers loaded with TCM ingredients, research on STCM-NDDS in cerebral ischemia therapy is still at a very early stage. However, several high-quality experimental studies have emerged in recent years. Table 1 summarizes carrier-free TCM supramolecular self-assemblies, and Table 2 presents carrier-loaded TCM nanoparticles (Tables 1, 2).

**TABLE 1:** Carrier-free STCM self-assemblies in cerebral ischemia treatment.

TCM component/source	Nanoparticle type	Assembly/preparation driving force	*In vitro* BBB penetration	Animal model	Administration route	Main efficacy	Ref.
Berberine-baicalin	Herb pair co-assembled NPs	Electrostatic, π-π, H-bond	Not verified	Mouse cerebral ischemia	Not reported	Anti-inflammatory, antioxidant	(Yi and Xu, 2004)
Quercetin-mecobalamin	Carrier-free co-assembled NPs	H-bond, hydrophobic	Enhanced *in vitro* model	Rat MCAO	Intravenous	Reduced oxidative stress, microglial modulation	(Zhang et al., 2026)
Ginsenoside Rb1 + 3-n-butylphthalide + probucol	Ternary carrier-free co-assembled NPs (SRPNNPs)	Hydrophobic, π-π	Polysorbate-80 enhanced	Rat CIRI	Intravenous	Synergistic anti-inflammatory, antioxidant	(Guo et al., 2025)
Naoluo Xintong decoction	Formula natural aggregates	Multiple weak bonds	*In vitro* penetration	Rat MCAO	Oral gavage	Anti-inflammatory, anti-apoptotic	(Zhao G. et al., 2022)

**TABLE 2:** Carrier-loaded TCM nanoparticles and TCM-derived nanoparticles in cerebral ischemia treatment.

Classification	TCM component/source	Nanoparticle type	Preparation strategy	*In vitro* BBB penetration	Animal model	Administration route	Main efficacy	Ref.
Carrier-loaded	Curcumin	Solid lipid nanoparticles (C-SLNs)	Lipid-embedded, low-temp solidification	Increased brain distribution	Rat MCAO	Intravenous	Inhibited AChE, improved oxidative stress	(Kakkar et al., 2013)
	Curcumin	Liposomes (CUR@LP-SHp)	Lipid encapsulation + FUS-opened BBB	Focused ultrasound-assisted	Photothrombotic stroke	Intravenous	92% infarct reduction	(Su et al., 2026)
	Puerarin	HP-β-CD-PLGA NPs	Cyclodextrin inclusion + PLGA	Significantly improved brain targeting	Rat MCAO	Intravenous	Anti-inflammatory, anti-apoptotic	(Tao et al., 2021)
	Tetramethylpyrazine	Solid lipid nanoparticles	Lipid embedding	Enhanced brain targeting	Rat MCAO	Intravenous	Anti-inflammatory, antioxidant	(Ji et al., 2017)
TCM-derived nanoparticles	Eupatorium lindleyanum	Carbon quantum dots (CQDs)	Pyrolytic carbonization	BBB penetration, wide brain distribution	Mouse cerebral ischemia	Intravenous	Targeting IDH1, inhibiting mtDNA-cGAS-STING	(Chen Z. K. et al., 2026)
	Crinis Carbonisatus (carbonized human hair)	Carbon quantum dots	Isolation	Reduced BBB permeability	Rat MCAO	Intraperitoneal	Reduced TNF-α, IL-6	(Zhang et al., 2021)
	Panax notoginseng	Exosome-like nanoparticles (ELNs)	Lipid-protein-nucleic acid natural complex	Transcytosis, ischemic aggregation	Rat tMCAO	Intravenous	Activated PI3K/Akt, induced M2 polarization	(Li S. et al., 2023)
	Angelica-Chuanxiong herb pair	Macrophage membrane-camouflaged biomimetic NPs	Oxalate ester-linked amphiphilic carrier + membrane coating	Macrophage membrane-mediated	Cerebral ischemia model	Intravenous	Synergistic infarct reduction by three components	(Su et al., 2022)

The references from 2025 to 2026 cited in the table are either online-first or recently published articles, all of which have undergone peer review or are currently in the formal publication pipeline.

Although the number of studies on strictly defined carrier-free STCM remains limited, the past 2 years have witnessed a clear growth trend in this area. Recent reports on quercetin-mecobalamin co-assemblies and ginsenoside ternary co-assemblies reported in 2025–2026 adopt ROS-responsive or active targeting strategies, yielding robust pharmacodynamic data in animal models. Meanwhile, research on TCM-derived carbon dots and exosome-like nanoparticles has generated a relatively systematic body of evidence supporting their efficacy in cerebral ischemia therapy. The study on *Eupatorium lindleyanum* carbon dots employed redox proteomics to identify IDH1 as the functional target, establishing a methodological paradigm for moving STCM from phenomenological description to mechanistic quantification. Studies on *Panax notoginseng* exosomes have progressed from initial BBB penetration and efficacy validation to engineered modification and microRNA loading.

Nevertheless, classic Qi-blood activating herb pairs such as Astragalus-Chuanxiong and Salvia-Notoginseng have not yet been reported for cerebral ischemia targeting using pure self-assemblies. The TCM formula Pushen capsule has shown therapeutic effects in ischemic stroke model mice by reducing cerebral infarct volume and improving BBB integrity; its aqueous extracts inherently contain supramolecular complexes (Zhang Y. et al., 2023).

Furthermore, rigorous comparative experiments that directly compare intact STCM with equivalent physical mixtures composed of unassembled monomers are currently lacking to verify that the multicomponent synergistic effect relies on specific supramolecular structures. Such experiments are critical to establishing the causal structure–function relationship of STCM and should be prioritized in future investigations.

## 5 Core challenges and future perspectives

Although STCM has demonstrated remarkable potential in the treatment of cerebral ischemia, significant challenges remain in translation from bench to bedside.

### 5.1 Chemical heterogeneity and batch-to-batch consistency

STCM is the collective behavior of multi-component dynamic libraries. Their critical assembly concentration, polydispersity, and metastable conformations are influenced by decoction temperature, time, and ionic strength. Current characterization of STCM structures predominantly relies on traditional methods (dynamic light scattering, TEM, UV spectroscopy), providing only bulk average information and thereby failing to establish a quantitative correspondence between supramolecular structure and chemical composition at the single-particle level. Compared with synthetic nanoassemblies, the multi-component nature of STCM presents greater challenges in terms of batch-to-batch consistency and structural validation. Synthetic nanoparticles typically consist of 1–2 highly purified materials, whereas STCM may involve the synergistic assembly of dozens of structurally similar active constituents. Batch-to-batch fluctuations in any single component may influence the overall assembly behavior.

Quality markers are a core concept in TCM quality control, which has traditionally relied on small-molecule compounds such as flavonoids, terpenoids, and alkaloids as quality indicators. In recent years, researchers have begun to explore the potential of utilizing STCM with stronger biological activity as novel quality markers, believing that supramolecular markers based on structure and activity could provide a new perspective for TCM quality control (Cheng et al., 2022). From a methodological perspective, supramolecular fluorescence sensing methods have been applied to evaluate the batch-to-batch consistency of TCM preparations. For instance, a sensor array based on calixarene was constructed to evaluate the batch-to-batch consistency and accuracy of raw material inputs of Yinxing Mihuan oral solution via fluorescence fingerprinting, providing a high-throughput technical solution for the quality control of TCM compound preparations (Niu et al., 2025).

### 5.2 The black box of biological fate

Upon entering the bloodstream, STCM rapidly adsorb a protein corona on their surfaces, altering their initial recognition properties (Monopoli et al., 2012). The composition and formation of the protein corona are governed by multiple physicochemical properties of the nanoparticles, including particle size, surface charge, and hydrophobicity. phobicity, and surface chemistry, which in turn influence their circulation time, tissue distribution, and cellular uptake *in vivo* (Chou and Lin, 2024; Guo et al., 2024). In the multi-component STCM system, the complexity of the protein corona is further amplified. Different components may competitively adsorb to different types of plasma proteins, leading to dynamic changes in the composition of the corona, which makes its *in vivo* fate difficult to predict.

Most studies track labeled STCM solely by fluorescence imaging, which cannot distinguish intact particles from free drugs. The distribution, metabolism, and clearance characteristics of these two states are distinct, yet the total signal provided by fluorescence imaging obscures this difference. Moreover, current research mostly focuses on the supramolecular structures formed by single herbs or single components, and there is still a lack of systematic understanding of the complex *in vivo* behavior of herb pairs or formula supramolecules and their dissociation-transcytosis-reassembly process at the BBB (Li et al., 2025).

The route of administration presents a further translational challenge. STCM is derived from oral herbal decoctions, but oral delivery requires nanoparticles to withstand harsh gastrointestinal conditions, including gastric acid, digestive enzymes, and microbial degradation. It remains unclear whether intact STCM nanostructures can reach the bloodstream and cross the BBB after oral administration. Injectable formulations avoid gastrointestinal barriers but introduce other pharmaceutical challenges, such as sterility control, pyrogen removal, and achieving uniform particle size distribution. All current STCM stroke studies adopt intravenous injection, confirming researchers' awareness of oral delivery limitations. We propose stage-specific administration strategies matched to STCM physicochemical properties. For the emergency intervention of acute stroke, intravenous formulations can be developed, and targeted formulation optimization can be adopted to overcome the obstacles related to sterility and particle size homogeneity. For long-term rehabilitation therapy post-stroke, oral enteric microcapsules are capable of resisting gastrointestinal degradation.

### 5.3 Causal gaps underlying drug delivery mechanisms

Most current research is limited to establishing correlations showing that STCM increases brain drug concentration and improves efficacy. However, whether the unique supramolecular structure itself generates new functions that cannot be reproduced by a mixture of free monomers, or whether it merely acts as a physical carrier to deliver active ingredients more efficiently to the target area, remains unresolved. To date, no study has employed rigorous comparative experiments to address this question, wherein pharmacodynamic comparisons can be performed between intact STCM and a physical mixture of chemically equivalent yet unassembled monomers. The core of this problem is that a convincing causal chain between supramolecular structure and function has not yet been established, and there is a lack of comparative experimental designs that strictly control variables to decouple the two.

Based solely on efficacy data, it is not possible to draw definitive conclusions about the fundamental drivers of treatment outcomes. Although monosaccharide-based nano-assemblies demonstrate exquisite supramolecular design combining reversible assembly and fluorescence modulation, the prospective analysis of that work pointed out that one of the key challenges for their translation to biomedical applications lies in the specificity of the response-triggering mechanism.

### 5.4 Future breakthrough directions

Regarding static structural analysis, current efforts to characterize STCM architecture predominantly rely on indirect methods. Cryo-electron microscopy (cryo-EM) can image samples at high resolution under near-native hydrated conditions and has become a mainstream tool in structural biology; it has also enabled significant breakthroughs in resolving atomic-level structures of soft materials such as supramolecular gels. Isothermal titration calorimetry can directly determine the binding constant, stoichiometry, and thermodynamic parameters of non-covalent interactions, and microscale thermophoresis can precisely measure molecular affinities in solution, both providing key data for the quantitative characterization of STCM assembly driving forces.

For dynamic process visualization, whole-brain clearing imaging and intravital two-photon microscopy can be further integrated to track the real-time transit of STCM across the BBB. Developing self-indicating STCM based on aggregation-induced emission (AIE) can yield distinct fluorescence responses for the assembled and disassembled states, thereby enabling visualization of *in vivo* fate and real-time assessment of structural integrity. AIE dye-based tracking of nanoparticle integrity serves as a promising strategy for distinguishing intact STCM from disassembled components, enabling in-depth exploration of the black box governing STCM *in vivo* fate.

Guided by computational supramolecular chemistry, establishing a framework for precision self-assembly represents a critical pathway to enhancing the controllability of STCM. By using computational chemistry and artificial intelligence to screen key molecular pairing candidates, researchers can predict the assembly driving forces and optimal stoichiometric ratios. Strategically, reversible covalent bonds (such as dynamic Schiff bases, boronate esters) may be introduced to lock the non-covalent network, improving structural stability without compromising responsiveness. A multi-parameter fingerprint quality control system incorporating particle size, polydispersity index, zeta potential, encapsulation efficiency, and key component ratios should be established. This pathway can provide a rational design framework for establishing the structure–function causal chain.

## 6 Conclusion

STCM is a natural nano-ordered aggregate that is widely present in TCM water decoctions. Research in this field is shifting the paradigm of TCM pharmacodynamic material bases from the single-ingredient view toward a systems perspective of ingredient-structure-function.

In this review, we have described the pathological changes in the BBB after cerebral ischemia and the low-pH, high-ROS, high-MMP microenvironment of the penumbra. We propose that these features provide the natural basis for responsive drug release by STCM. Based on this, we comprehensively analyzed the unique advantages of STCM in efficiently crossing the BBB and precisely delivering drugs via supramolecular multivalent surfaces, the integration of aromatic resuscitation components, the dissociation of non-covalent networks, and the structural division-functional synergy of herb pair components.

Although current STCM research faces significant challenges, such as chemical heterogeneity, unclear *in vivo* fate, and gaps in establishing causal drug delivery mechanisms, the advent of cryo-EM, AI-assisted design, multimodal *in vivo* imaging, and precision self-assembly technologies positions STCM as a potential disruptive platform for precision-targeted therapy of cerebral ischemia. Future efforts should focus on establishing *in situ* characterization methodologies for STCM, crossing the three major thresholds from phenomenological description to mechanistic quantification and then to clinical translation, so that this nanomedicine strategy rooted in the wisdom of traditional Chinese medicine can truly benefit patients with cerebral ischemia.
